# Sensory TRP Channel Interactions with Endogenous Lipids and Their Biological Outcomes

**DOI:** 10.3390/molecules19044708

**Published:** 2014-04-15

**Authors:** Sungjae Yoo, Ji Yeon Lim, Sun Wook Hwang

**Affiliations:** Department of Biomedical Sciences, Korea University College of Medicine, Seoul 136-705, Korea; E-Mails: headyoo@korea.ac.kr (S.Y.); ljyangel1004@korea.ac.kr (J.Y.L.)

**Keywords:** sensory TRP ion channels, lipids, C-fibers, pain, inflammation

## Abstract

Lipids have long been studied as constituents of the cellular architecture and energy stores in the body. Evidence is now rapidly growing that particular lipid species are also important for molecular and cellular signaling. Here we review the current information on interactions between lipids and transient receptor potential (TRP) ion channels in nociceptive sensory afferents that mediate pain signaling. Sensory neuronal TRP channels play a crucial role in the detection of a variety of external and internal changes, particularly with damaging or pain-eliciting potentials that include noxiously high or low temperatures, stretching, and harmful substances. In addition, recent findings suggest that TRPs also contribute to altering synaptic plasticity that deteriorates chronic pain states. In both of these processes, specific lipids are often generated and have been found to strongly modulate TRP activities, resulting primarily in pain exacerbation. This review summarizes three standpoints viewing those lipid functions for TRP modulations as second messengers, intercellular transmitters, or bilayer building blocks. Based on these hypotheses, we discuss perspectives that account for how the TRP-lipid interaction contributes to the peripheral pain mechanism. Still a number of blurred aspects remain to be examined, which will be answered by future efforts and may help to better control pain states.

## 1. Introduction

**Figure 1:**
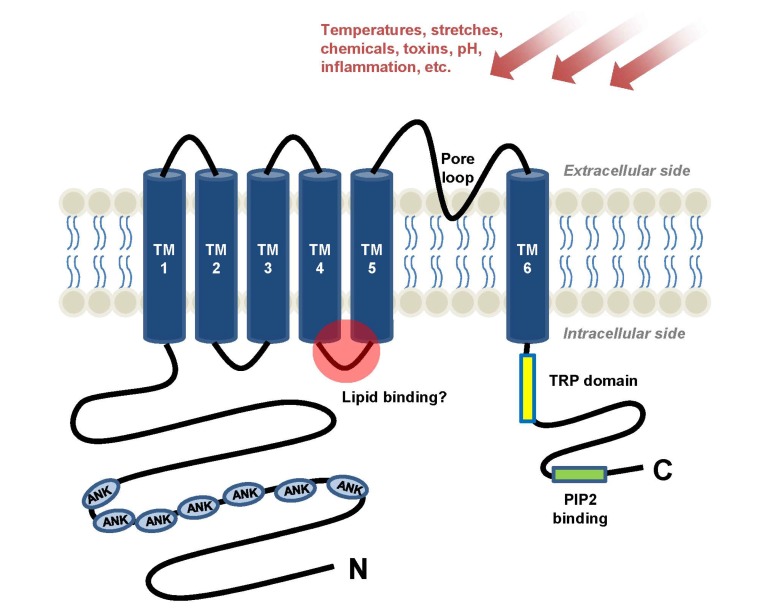
A topological structure of sensory TRP channels is illustrated. A subunit of the sensory TRP channels has six membrane-spanning (transmembrane) domains. Between TM5 and TM6 domains, the pore loop that allows ionic flow is located. Amino and carboxyl termini are located in the cytosol. Some of sensory TRPs contain multiple ankyrin repeats at their amino termini. One of important locations to interact with endogenous lipid regulators or lipophilic pharmacological agents (*i.e.*, capsaicin) is predicted to be the TM4eTM5 linker. PIP2 may binds to C-termini of some TRP channels near the TRP domain.

Perception of external and internal stimuli is an essential function of the brain for adaptation, avoidance and protection. Perception of environmental changes is prompted by their detection by somatosensory primary afferents, namely the dorsal root ganglion (DRG) neurons for the body and trigeminal neurons for the face. Sensory axons terminate at the skin epidermal or dermal areas to sense changes that occur near our body. When environmental changes are strong enough to overcome thresholds, action potentials are ignited in the peripheral axon termini, and the electrical discharges travels up to higher brain regions such as the somatosensory cortex, resulting in perception. A subset of the sensory afferents is responsible for recognizing painful (or potentially damaging) changes that finally evoke pain perception: unmyelinated small-diameter C-fibers and thinly-myelinated Aδ fibers. Some of TRP ion channels are important molecular sensors that those fibers contain, for the detection of damaging attacks (Figure 1). Environmental changes can be subcategorized as mechanical, thermal and chemical changes, or in another way, as external and internal ones. Chemical changes, particularly, which includes substances released near sensory nerve terminals, can encompass the majority of internal environmental changes. Examples of those substances include cellular components like nucleotides or peptides released from dying tissues, neurotransmitters, pro-inflammatory mediators, or specific lipids that can interact with molecular sensors in sensory fibers.

Mammals, including humans, express 28 to 29 TRP ion channels. As mentioned above, several of these TRPs serve as sensors in the nerve terminals of C-fibers and Aδ fibers. These TRPs open their pores in response to relevant stimuli, which depolarizes the nerve to produce action potentials. Interestingly, all of these sensory TRP channels are polymodal to some extent. First, they are all sensitive to a range of temperatures and some of sensory TRPs respond to mechanical insults. With respect to chemicals, a surprisingly large number of substances including different types of lipids, can activate sensory TRP channels. Given their high degree of polymodality, sensory TRP channels are believed to act as major sensor molecules sending signals regarding environmental risks to the brain in the form of pain perception. While such roles are central for signal generation during the alert states, the painful aspect often emphasizes TRP pharmacology for the purpose of analgesia [1]. Lipid ligands are sometimes highlighted in such research because they may offer a starting place for good synthetic leads and, at the very least, be a key molecule for understanding the unknown mechanisms of peripheral pain. In this review, we introduce the sensory roles for each type of sensory TRP channel (TRPV1-V4, TRPA1 and TRPM8), discuss their interactions with lipids, and provide our perspectives on future research directions.

## 2. Roles of Sensory TRP Channels in Pain Sensation

### 2.1. Vanilloid Subtype 1 (TRPV1)

TRPV1 is a central sensor molecule that exibits polymodality. TRPV1 opens in response to noxious heat (>42 °C), protons, lipidergic pungent chemicals or polyamines [2,3,4,5]. In addition, hypertonicity has been shown to induce TRPV1 activation [6]. TRPV1 activation in sensory nerve termini leads to neuronal excitation, which ultimately causes pain perception as described above. The painful hot sensation of red peppers results from direct TRPV1 binding by capsaicin, a main pungent ingredient. Exacerbation of tissue inflammation and inflammatory pain also involves sensory neuronal TRPV1 activation. Major inflammatory mediators such as nerve growth factor, bradykinin, and prostaglandins excite and sensitize C-fibers by elevating TRPV1 activity via metabotropic receptor signalings [7]. Also, protons and lipids such as leukotrienes from inflamed or damaged cells directly activate TRPV1. Inflamed or injured situations always form “calor” (increased heat) conditions and sometimes form a hypertonic or acidic environment, which indicates that multiple stimuli such as heat, proton abundance, hypertonic stress, and lipids may cooperatively synergize TRPV1 activation even when the individual stimuli are below the necessary threshold [8,9,10].

### 2.2. TRPV2

The existence of a sensory component that detects noxiously high temperatures has been raised in medium to large diameter DRG neurons [11]. TRPV2 channel is found to be the component that opens in response to noxious heat and has a threshold of 51 °C. TRPV2 is expressed in thinly myelinated sensory afferents with medium to large size soma [12,13,14,15,16], a subset of which also expresses TRPV1 [16,17,18]. In the complete Freund’s adjuvant (CFA) inflammation model, TRPV2 expression is heightened and it contributes to heat hyperalgesia [19]. TRPV2 chemical sensitivity occurs under the same inflamed condition [20]. Mechanosensitivity of TRPV2 has also been suggested. [21,22,23,24]. However, genetic approaches often failed to confirm the mechanical phenotype. An *ex vivo* study using TRPV1-deficient DRG neurons cast doubt on TRPV2’s role in acute heat nociception [25]. Likewise, TRPV2-null mice exhibit no difference from their wild-type littermates with respect to heat and mechanical nociception [26].

### 2.3. TRPV3

TRPV3 is also a heat-activated channel with a threshold of 33 °C [27,28,29]. Different from other sensory TRPs, TRPV3 is mainly expressed in epidermal keratinocytes [27,29,30,31]. Accordingly, the epidermal layer is considered as an outpost sensor organ. TRPV3 activation may evoke intercellular transmitter secretion from keratinocytes, which in turn are detected by neighboring C-fiber termini to result in depolarization. TRPV3 knockout mice and overexpressing transgenic mice proved TRPV3 as a heat pain sensor [30,32,33]. TRPV1 and TRPV3 share their sensing temperature bands (greater than 42 °C), although TRPV1 plays the major thermonociceptive role. TRPV3 is presumed to have developed evolutionarily as a backup heat pain sensor [32,34]. Surprisingly, firm evidence that TRPV3 is important for pain sensation has accumulated in industry research. Preclinical and clinical trials of synthetic TRPV3 antagonists from Hydra Biosciences, Sanofi-Aventis, GlaxoSmithKline, and Novartis have demonstrated promising analgesic results in a number of of inflammatory and neuropathic pain models [35,36,37,38,39].

### 2.4. TRPV4

TRPV4 is a polymodal ion channel sensitive to heat (with a threshold of 27–34 °C), chemicals, and mechanical insults, including hypotonicity [40,41,42]. Mechanosensitivity seems to be the most obvious modality of TRPV4 considering these related pain phenotypes. Using TRPV4-null mice, the Levine lab showed that TRPV4 mediates acute inflammatory mechanical hyperalgesia [43,44]. In addition, the signaling of protease-activated receptor 2 (PAR2), which is a G-protein coupled receptor (GPCR), utilizes TRPV4-mediated substance P and calcitonin gene-related peptide (CGRP) secretion in the spinal cord dorsal horn, which enhanced mechanical hyperalgesia [45]. TRPV4 expression levels in thoraco-lumbar colonic DRG neurons or wild types were higher than in whole ganglia and TRPV4 knockouts exhibit blunted responses to noxious colonic distension [46,47]. Further, specific TRPV4 modulators and serotonin or histamine could control TRPV4-mediated visceral mechanosensitivity, indicating that TRPV4 may contribute to mechanically evoked visceral pain [46,47,48]. TRPV4 potentiaion by serotonin was consistently observed in other tissues [49]. In neuropathic conditions including a rat chemotherapy-induced neuropathy model and a chronic constriction injury model, TRPV4 was shown to contribute to mechanical hypersensitivities [50,51]. For thermal pain, TRPV4-knockouts exhibit attenuated acute thermal pain [52], reduced inflammatory thermal hyperalgesia and decreased neuronal firing in response to warm temperatures [53].

### 2.5. Ankyrin Subtype 1 (TRPA1)

TRPA1 is a popular analgesic target because of its multi-modal nature. Owing to its chemical sensitivity to a large number of heterogeneous cellular components including lipid substances as described below, TRPA1 is called a “cellular damage-sensing ion channel”. Still, the pool of TRPA1 activators continues to grow [54,55]. With respect to physical sensations, TRPA1 covers noxious cold temperatures (<17 °C) and noxious mechanical stretching [56,57,58,59,60,61,62,63]. TRPA1 also appears to play a role in cold sensing in the context of injury-related pathological pain, different from the function of TRPM8 mentioned below. TRPA1 is expressed in a subset of TRPV1-positive C-fibers, which indicates that that subpopulation composes namely “polymodal nociceptors” because the two TRPs covers extreme polymodalities. Furthermore, TRPA1 is comparable to TRPV1 in that they both serve downstream effector roles for pro-inflammatory mediators actions, the final result of which is inflammatory pain. Bradykinin, proteases and nerve growth factor (NGF) all utilize TRPA1 as their downstream [60,61,64,65,66]. All those indices on TRPA1 functions consistently highlight its role as a major pain sensor comparable to TRPV1 [67].

### 2.6. Melastatin Subtype 8 (TRPM8)

TRPM8 is a cold-activated channel (<26 °C) [68,69,70]. Under normal conditions, TRPM8 in mammals is thought to be responsible for detection of unpleasant, cold temperatures. Unlike the extended polymodal nature of TRPA1-mediated nociception, TRPM8 has been shown to be selectively involved in aberrant cold sensitivity in case of chronic pain [71,72,73,74,75,76,77]. Despite evidence that TRPM8 mediates cold-associated pain, it is disputable whether TRPM8 is a good analgesic target. For example, injection of TRPM8 activators relieves inflammatory and neuropathic pain in a TRPM8-dependent manner, and also inhibits TRPV1/TRPA1-mediated chemosensory and mechanosensory responses from visceral afferents [72,78]. In addition, in the acute phase of formalin-induced pain, cold-induced analgesia was observed in wild type mice but not in TRPM8-deficient mice [77].

### 2.7. Neurogenic Inflammation Mediated via TRP Activation

Nociceptive C-fibers comprise peptidergic and non-peptidergic populations. Persistent excitation of the peptidergic subset, for example by depolarization via sustained TRP activation caused by successive harmful input from neighboring inflamed tissues, results in not only nerves sending impulses to higher synapses for pain perception, but also causes nerve termini to release neuropeptides including CGRP and substance P into the inflamed tissues in retrograde fashion: eventually this begins to form a vicious signaling circuit. Although most studies on this circuit have focused on TRPV1, other TRP channels expressed in peptidergic C-fibers such as TRPA1 may also be closely involved [7]. Neuropeptides induce a more inflammatory state in the tissues and cause the tissues and infiltrated immune cells to secrete stronger stimulatory signals toward TRP channels and other receptors of peptidergic C-fibers. Moreover, CGRP and substance P also promote the function of C-fibers in an autocrine manner [79,80,81,82,83]. Thus, these neuropeptides aggravate inflammatory states and pain states. In this way, inflamed tissues and immune cells repetitively stimulate C-fibers more, by generating a larger amount of mediators. This circuit is called neurogenic inflammation [84]. This hyperactivation signal often strengthens central synaptic plasticity, which may provide a mechanism for chronic pain.

## 3. Endogenous Lipid Interaction of Sensory TRPs

**Figure 2:**
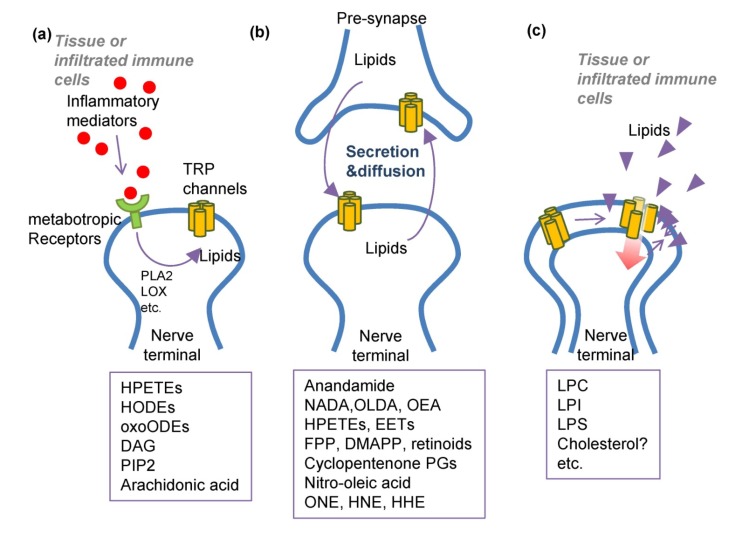
Three possible modes of interactions of TRP channels and their lipid modulators. (**a**) Traditionally, lipid modulators have been presumed as second messengers for signal transduction evoked by other stimuli; (**b**) Roles of synaptic neurotransmitters or intercellular transmitters are recently being raised; (**c**) Lipid modulators would alter the plasma membrane structure, leading to changes in TRP channel gating.

Information on the interactions between endogenous molecules can offer mechanistic insights into biological processes. Such information regarding sensory TRP channel interactions with lipids may shed light on pain states. Moreover, structural and biosynthetic information of lipids can be utilized to generate schemes for creation of TRP channel modulators. The history of lipid studies in the TRP field is relatively short starting in the late 1990s [85,86,87]. Nonetheless, knowledge has so far been actively expanding at various aspects such as ligand binding, sensitivity shift, bilayer-protein interactions, and upstream metabotropic signalings and so forth. However, perspectives were not settled down distinguishing whether TRP-reactive lipids act as second messengers, intercellular transmitters, or function to simply transform the plasma membrane properties affecting channel protein structure. Channel-modulating lipids were initially considered to act only as second messengers [88,89,90,91,92,93]. With respect to sensory TRP channels, this tradition still remains important to create hypotheses regarding pain deterioration [64,94]. However, in addition to the second messenger aspects, we also reviewed interactions from other viewpoints which have recently matured in the literature (Figure 2).

### 3.1. Second Messenger Hypothesis

It has only been several years since lipids were first hypothesized as general second messengers in intracellular signal transductions before ion channel-lipid study was born [95,96,97]. In the mid-1980s, K^+^ channels were reported in regards to the lipid effect as mentioned above. Some paralogs of lipid-gated two pore K^+^ channels are now also known to be related to pain sensation [98]. Na^+^ channel studies started in the same year but researchers approached the interaction with a more structural focus [99,100]. Cl^−^ channel joined it a few years later [101,102]. A seminal report on TRP channels was released in 1999. Hardie and colleagues found that TRP and TRPL of the fruit fly *Drosophila* are opened by polyunsaturated fatty acids (PUFAs) [85]. In the same year, Högestätt and his collaborators showed that a sensory TRP channel, TRPV1, is activated by an endogenous cannabinoid anandamide (*N*-arachidonyl ethanolamine, AEA) [86].

#### 3.1.1. PLA2-LOX Metabolites

**Figure 3:**
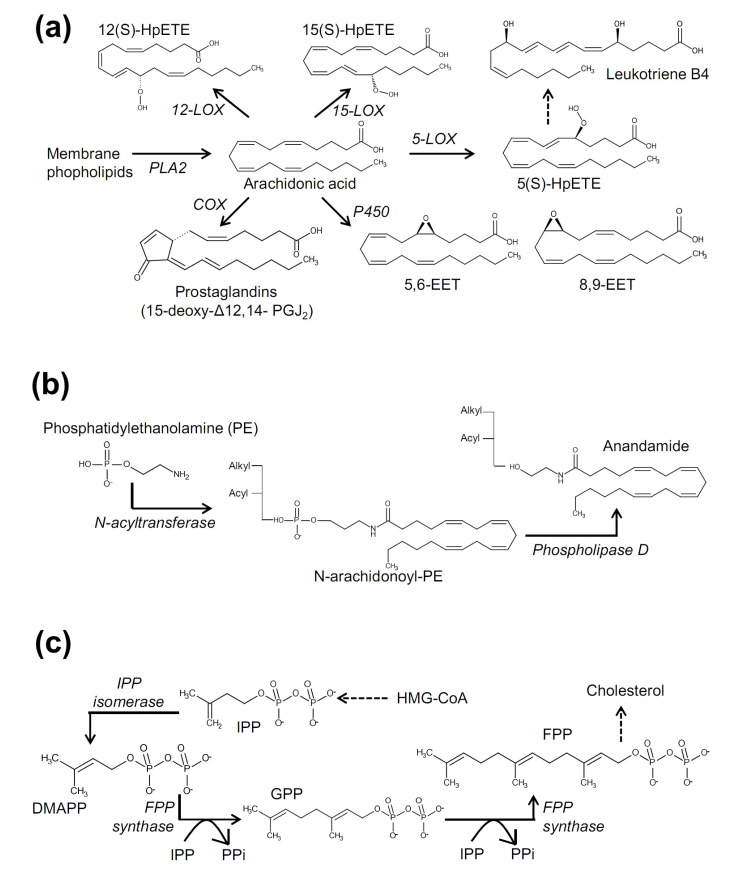
Biosynthetic pathways for HpETEs, EETs (**a**); anandamide (**b**); and isoprenoids (**c**), that are known to regulate activities of sensory TRP channels.

Since the 1999 report from Zygmunt *et al.*, a number of endogenous lipids have been investigated as TRPV1 channel activators: Lipoxygenase (LOX) metabolites are representatives of this class of molecules. Intracellular signaling evoked by exposure to bradykinin, a pro-inflammatory mediator, leads to LOX metabolite production inside C-fibers [94]. Among the resulting metabolites, 12(*S*)-hydroperoxytetraenoic acid (12(*S*)-HpETE) and 15(*S*)-HpETE potently activate TRPV1 [87,103] (Figure 3). This is interesting since one of the historically important lipids in traditional lipid-channel interaction studies at a viewpoint of second messenger hypothesis was also HpETEs. HpETEs were shown to activate *Aplysia* K^+^ channels, which constitute the downstream mechanism of a neuropeptide Phe-Met-Arg-Phe-NH_2_ (FMRFamide) signaling [88]. In mammalian sensory physiology, NGF, prostaglandins (PGs) and bradykinin are the three major inflammatory pain mediators via affecting C-fiber functions, which often leads to hyperalgesia. NGF and PGs are known to only sensitize sensory excitability, rather than directly eliciting neuronal firing. A proposed mechanism for these two mediators states that the increased abundance of TRPV1 channel proteins on surface membranes is due to its upregulated trafficking or TRPV1 protein is phosphorylated [104,105]. In contrast, bradykinin is able to evoke generation of action potentials, which utilize HpETEs [94]. Specifically, B2 type GPCR bound to bradykinin in C-fiber termini results in downstream activation of a Gq protein cascade. Accordingly, phospholipase A2 (PLA2) is activated to produce free n-6 arachidonic acid from the plasma membrane lipid bilayer. LOX then adds oxygen molecules to multiple carbons of arachidonic acid and as a result, intracellular concentrations of 12(*S*)-HpETE, 15(*S*)-HpETE are increased. Eventually, these oxygenated eicosanoids bind to and open TRPV1, which is typical of the activity of other second messengers on ion channels. Further reduced forms such as 5-HETE and 15-HETE and leukotriene B4 (LTB4), a 5-HETE metabolite are also able to activate TRPV1 [87] (Figure 2).

Although all of the known sensory TRPs are sensitive to particular ranges of temperatures, the mechanism of temperature-induced channel activation is not well understood. Interestingly, the Hargreaves lab suggested a lipid second messenger hypothesis for heat activation of TRPV1 [106]. In fact, the leading theory for this heat activation is that a specific domain or sequence of amino acids on TRPV1 protein may sense the elevation of temperatures, which leads to TRPV1 pore opening. For example, the Latorre group proposed that TRPV1’s direct heat-sensing domain is located in the *C*-terminal (and TRPM8’s cold-sensing domain, too) which they determined using domain-swapping assays [107,108]. Other specific amino acids in the pore region were also found to be important in heat activations of TRPV1 and TRPV3 [109]. A thermodynamic-based hypothesis has also been proposed that a relatively broader region may cooperatively contribute to the direct temperature sensation [110]. However, Hargreaves and colleagues argued that LOX metabolites are responsible for this heat activation. Specifically, when sensory neurons are exposed to heat, hydroxyoctadecadienoic acids (HODEs: 9-HODE and 13-HODE) and their further metabolized products oxoODEs are generated in the cytosol and then those lipid substances activate TRPV1 by binding inside. Other heat-sensitive TRPs such as TRPV2, V3 and V4 were not affected by the presence of these lipids. It is still not clear whether HODEs or the substrate linoleic acid are produced enzymatically (via LOX or PLA2) or non-enzymatically [111]. Future studies are needed to clarify which direct heat sensing or heat-generated lipid is the major mechanism for TRPV1 activation.

Lessons from bradykinin-TRPV1 coupling studies inspired the quest to identify lipid second messengers that activate another highly nociceptive TRP, TRPA1. As a result, bradykinin B2 receptor downstream is found to utilize both PLA2 and phospholipase C (PLC) for TRPA1 opening. Upon B2 receptor activation, arachidonic acid and diacylglycerol (DAG) are produced intracellularly via the action of these two enzymes respectively. Eventually, arachidonic acid and DAG can open TRPA1 [64]. Different from the case of TRPV1 where further metabolism is required to generate HpETEs, arachidonic acid seems to directly activate TRPA1. DAG may need additional steps. DAG can be degraded into polyunsaturated fatty acids (PUFAs) including arachidonic acid and then they may open TRPA1, or alternatively, activate kinases for TRPA1 phosphorylation, which results in greater activation.

#### 3.1.2. Lipids in the PLC Cascade

DAG has been actively studied as an activator for canonical types of TRP (TRPC) channels. Oh and colleagues demonstrated that DAG also activates TRPV1 [112,113]. Different from the case of TRPA1 described above, Oh’s group showed that direct binding of DAG may open TRPV1 by excluding the possibility that downstream of DAG signaling such as phosphorylation by DAG-activated kinase is uninvolved. Another interesting finding from the same lab is that a TRPV1 mutant with impaired capsaicin binding loses its ability to be activated by DAG. This implies that capsaicin and DAG may share a common TRPV1 binding site. This DAG activation of TRPV1 may account for mGluR5 signaling in the central termini of C-fibers in the lamina II spinal cord layer [113].

Phosphatidylinositol 4,5-bisphosphate (PIP2) is a lipid component found primarily in the plasma membrane inner layer. PLC hydrolyzes PIP2 into DAG and inositol 1,4,5-trisphosphate (IP3). PIP2 is known to regulate a diverse set of ion channel functions via direct contact [114]. For TRP channel species, PIP2 binding tends to contribute to channel activation. PIP2 depletion downregulates activities of many TRP channels including TRPV1, TRPV2, TRPV5 TRPM4, TRPM5, and TRPM7 [115] of which, the cold receptor TRPM8 is the best example [116,117,118]. PIP2 binds to the TRP domain located in the *C*-terminal of TRPM8, causing TRPM8 activation. Basic amino acids conserved throughout the majority of TRP channels is defined as TRP domain, which may account for the less selective actions of PIP2 on a multitude of TRP species. PIP2 binding can promote TRPM8 sensitivities to cold and menthol. Interestingly, exposure to cold temperatures or menthol also raises the responsiveness of TRPM8 to PIP2, indicating that stimulation by these three are mutually synergistic. Since the PIP2 binding site and the cold-sensing domain are located in separate regions of the protein, PIP2 is likely uninvolved in the cold activation mechanism of TRPM8, but may exhibit allosteric effects [108].

PIP2 is present in most cell types and is a common signaling molecule, the levels of which are regulated by the signal transduction enzyme PLC. This may lead to a notion that TRP channels are tonically open owing to the presence of PIP2 and that a cellular event that activates the PLC pathway may close the channel via PIP2 consumption. Indeed, TRPM8 is desensitized upon increased cytosolic Ca^2+^ that enters the cell through the open pore of TRPM8 itself. It is likely that activated PLC by Ca^2+^ cleaves PIP2 bound to TRPM8 [116,117]. Mercado *et al.* found that a similar mechanism works for TRPV2 modulation. PIP2 depletion by Ca^2+^-activated PLC accounts for Ca^2+^-dependent desensitization of TRPV2 [119]. In addition, incorporation of extra PIP2 in cells is able to protect TRPV2 from desensitization to some extent. Direct internal binding has also been proposed similar to other TRPs. However, the location of its binding site in TRPV2 remains unclear since the known PIP2-binding site is not conserved.

The direction of the effect of PIP2 is unpredictable in some TRP channels. Presence of PIP2 is able to interfere with TRPA1 activation by other agonists, and PIP2 sequestration promotes TRPA1 activation [120,121]. Noguchi and colleagues demonstrated that the PAR2 receptor pathway employs this mechanism, namely, decreasing levels of PIP2 to achieve TRPA1 sensitization, which deteriorates behavioral nocifensive responses [120]. This “dis-inhibition” mechanism was also observed in the bradykinin-PLC-TRPV1 axis, although there have been conflicting results as briefly mentioned above [104,122]. Similar to HpETEs, PIP2-mediated signal transduction may also be important for bradykinin-induced TRPV1-specific pain. It is plausible that two different target sites for PIP2 may explain these contradictory actions on TRPV1 [108,122,123,124]. Rohacs and his colleagues suggested that opposite actions of PIP2 may depend on the extent of TRPV1 activation [125]. Also, one of these actions may involve indirect signaling, rather than direct binding of PIP2.

### 3.2. Intercellular Transmitter Hypothesis

This viewpoint is that sensory TRP channels act as ligand-gated channels and that we see lipids as their ligands. Although many of the identities of TRP ligands overlap with those listed above for second messengers, there exists additional evidence suggesting that lipids can act as neurotransmitters (or intercellular transmitters in case of non-neuronal cells) [86,126,127]. Experimental validation of this hypothesis, however, remains less complete particularly with respect to conventional criteria for defining neurotransmitters, namely: (1) the substance is secreted from the presynaptic cell; (2) the presynaptic cell is confirmed to express the necessary metabolic enzymes to produce the substance; (3) the amount of the secreted substance readily reaches the receptor threshold; (4) The interaction between the substance and the postsynaptic receptor is specific; and (5) the presynaptic cells have the transportation machinery necessary for substance reuptake to halt the substance’s action and to recycle the substance. Numerous studies have tried to find evidence for (1), (3), and (4), but few have focused on (2) and (5).

#### 3.2.1. Amine Conjugates of Fatty Acid

Amine conjugates of fatty acids appear to be treated according to the neurotransmitter standard. This view may have originated from conventional anandamide research, where this substance has long been studied as a cannabinoid receptor agonist (Figure 3B). With respect to chemical structure, amine conjugates have a relatively clear polar region compared to those of LOX metabolites in which oxygenated carbons are scattered along the aliphatic chains. Amine conjugates possess an amide bond or that with a guaiacolic or a catecholic ring. This combined structure resembles that of capsaicin, which comprises a carbon chain, an amide bond and a guaiacolic aromatic ring, and this similarity may allow the amine conjugates to access TRPV1’s capsaicin-binding pocket.

Members of the amine conjugate category that are known to activate TRPV1 include anandamide, *N*-arachidonoyl dopamine (NADA), and *N*-oleoyl dopamine (OLDA), and *N*-oleoyl ethanolamine (OEA) [86,126,128,129,130]. Interestingly, not only anandamide, NADA also activates cannabinoid CB1 receptor [126,131]. Cannabinoid receptors may also require this structural property. Such multiple actions on heterogeneous receptors often lead to difficulties in interpretation of *in vivo* behavioral results. Specifically with respect to pain, the outcomes for TRPV1 and CB1 activations *in vivo* are exactly the opposite (as known very well, cannabinoid injections relieve pain). It would also be interesting to determine which receptors dominate changes in the cellular function caused by these amine conjugates, even in one neuron, since a subset of C-fibers and hippocampal neurons co-express CB1 and TRPV1 [132,133]. Recently, anandamide is shown to be produced in C-fibers and behave in an autocrine manner, which is hypothesized to result in amplification of excitatory signals of C-fibers due to TRPV1 activation rather than its attenuation due to CB1 activation [134,135].

Locations for neurogenic inflammation are not limited to the somatosensory dermatome. It occurs in the vascular system where fatty acid amides are known to play a key role. Vascular endothelia are known to release anandamide. TRPV1-expressing afferents innervate blood vessels. When the vascular anandamide opens neuronal TRPV1, nerve endings may secrete CGRP onto the blood vessels, causing potent vasodilation [86,136]. If this signaling cycle becomes intensified in the meningeal region, it may result in a headache.

Hypersensitivity is one of major symptoms of psoriasis, in which epidermal arachidonic acid levels can be elevated up to 10–100 µM [137]. Hu and his colleagues hypothesized that the activity of TRPV3, an epidermal sensory TRP channel, can be affected by this arachidonic acid-rich condition. They found that arachidonic acid and some other PUFAs are able to sensitize TRPV3 activity [137]. This sensitization cannot be replicated in TRPV1 or TRPV2 experiments, and thus it is TRPV3-specific. The Hu lab also found that anandamide sensitizes TRPV3 activity, while DAG is inert to TRPV3 activity. It seems that anandamide is converted into arachidonic acid and then arachidonic acid acts on TRP channels as previously shown in a TRPV4 study [138]. Further metabolic conversions may not be required according to data from the Hu lab using non-metabolizable analogues, which is reminiscent of the findings for TRPA1 described by Bandell *et al.* [64].

#### 3.2.2. Hydroperoxy Eicosanoids

Above, we discussed internal generation of LOX metabolites as second messengers under control of upstream receptor signaling. This may occur in tissues surrounding C-fiber endings, infiltrated monocytes or neutrophils in the presence of inflammation or injury [139]. These substances can be excreted and diffuse around nerve endings like any other transmitter or mediator. It is also possible that the substances may flood the nerve so as to affect the nerve itself again or the neighboring terminal branches, which is indicative of a typical autocrine or paracrine effect. This kind of intercellular communication may contribute to exacerbation of TRPV1-related pain.

The neurotransmitter-like actions of lipidergic TRPV1 ligands have been demonstrated in studies on central synapses. Initially, Gibson *et al.* reported the release of 12(*S*)-HpETE in the hippocampal synapses and its presynaptic TRPV1-mediated outcomes for long-term depression (LTD) [127]. Since then, a series of studies on anandamide have confirmed its role as a lipid transmitter to regulate pre- and post-synaptic TRPV1 function for LTD development in the dentate gyrus, nucleus accumbens, and hippocampus [140,141,142]. NADA is also known to be present in the central nervous system [126]. Depending on the specific localization of TRPV1 among central synapses, it is very probable that TRPV1 is not a nociceptive sensor, but that heterogeneous roles for the lipid-TRPV1 interaction out of pain might be detected, for example, learning and memory, emotion, *etc.*

With respect to the spinal synapses, previous studies have tested the pain-focusing concept. The Hargreaves group actually raised a centrally expressed TRPV1-related mechanism for allodynia development by emphasizing that HODEs and oxoODEs, the peripheral heat signal downstream molecules mentioned above, are also formed in the spinal cord upon depolarization [143]. Different from its potential role as a second messenger in the periphery, since it is impossible for the spinal cord region to experience temperature elevation up to a noxious range (>42 °C), these molecules might be synthesized following some specific synaptic signals in order to mediate autocrine or intercellular communications.

Intramolecular rearrangement of 12(*S*)-HpETE can generate epoxy metabolite species such as hepoxilins. A study performed by Yaksh and his colleagues demonstrated that hepoxilins activate TRPV1 and TRPA1 located in the central terminals of C-fibers, which leads to a hyperalgesic state [144]. Neighboring cells, but not C-fibers themselves, are predicted to be the source of hepoxilins. Hepoxilins are more chemically stable than HpETEs. Furthermore, the breakdown processes for HEPTEs and hepoxilins seem to be different: enzymes in charge of the breakdown of 12-HEPTE and hepoxilin A3 are glutathione peroxidase and epoxide hydrolase, respectively [145]. It is unclear whether hepoxilins also work in the periphery like HpETEs. Therefore, despite sharing a chemical origin and final biological outcome (pain), HpETEs and hepoxilins might play differential roles depending on their location and duration which may result in different severities. In addition, future studies on the spinal hepoxilins could more delicately define difference resulting from TRPV1 or TRPA1 activation at central termini. Studies regarding the possibility that hepoxilins can function as second messengers under control of some other neurotransmitter are also necessary.

#### 3.2.3. Epoxy Eicosanoids

Humans have three major enzymes for arachidonic acid oxidation: LOX, cyclooxygenase (COX), and cytochrome P450 epoxygenase. Epoxyeicosatrienoic acids (EETs) are the products of the P450 epoxygenase pathway (Figure 2A). Putative substances (namely, endothelium-derived hyperpolarizing factors; EDHFs) that cause vasodilation by activating smooth muscle Ca^2+^-activated K^+^ channels were now known to be EETs [146,147,148]. Interestingly, TRPV4, which is highly Ca^2+^-permeable, is expressed in the vascular endothelium linings [149,150]. Presence of EETs and TRPV4 in the same region, the importance of Ca^2+^ in functions of these two ion channels, and lipid lessons from other TRP channels may lead the Nilius group to hypothesize that all of these activities may be connected. Importantly, they demonstrated that it is indeed the case: 5,6-EET and 8,9-EET open TRPV4 channels in a membrane-delimited manner [138,151]. TRPV4 seems to contribute to EETs’ action on blood vessel relaxation by supplying Ca^2+^ to the K^+^ channels [152]. Quantitative translation of TRPV4 actions, combining the final outcomes from Ca^2+^-activated K^+^ channel action may still be needed. Anandamide has also been reported as a vasodilator [153]. It is likely that anandamide is metabolized into EETs by an epoxygenase, and that EETs act on TRPV4 [138]. In the somatosensory field, TRPV4 has been known to be intrinsically osmo- and mechanosensitive, but it would be worth testing EET involvement for the gating mechanism and further, whether this may be related to hypotensive or hypertensive states in the vascular system [154]. However, P450 upstream signaling or pain mediation regarding EETs’ actions on TRPV4 remain less explored. Very recently, PAR2 was found to employ the EET production mechanism for TRPV4 activation [155].

Epoxygenases and EETs are present in trigeminal neurons, and tonic release of EETs has been shown to aid CGRP secretion upon neuronal excitation [156]. EET production is also detected in DRG and dorsal horn neurons [157], where the neuronal target of EET was TRPA1. TRPA1 present in DRG central termini also seems to serve a role for relaying mechanical allodynia signaling. The mechanism of effect consists of 5,6-EET enhancing spontaneous excitatory postsynaptic currents (sEPSCs), which depends on TRPA1. Future studies will determine whether autocrine, paracrine, or antidromic effects dominate for EET-TRPA1 interactions.

#### 3.2.4. Isoprenyl Substances

Plants produce isoprene lipids (terpenoids) for a variety of purposes. Many of those have already been shown to open TRPV3 [30,31,158,159,160]. Mammals also produce isoprene derivatives, and so far farnesyl pyrophosphate (FPP) has been identified as a TRPV3 activator [161] (Figure 3). The mammalian isoprene metabolic process is known as the 3-hydroxy-3-methylglutaryl-CoA (HMG-CoA) reductase pathway or mevalonate pathway (or cholesterol synthesis pathway, as one of its famous final products is cholesterol) (Figure 3C). A series of acetyl-CoA condensation generates HMG-CoA. HMG-CoA reductase using nicotinamide adenine dinucleotide phosphate (NADPH) reduces HMG-CoA into mevalonate. Subsequent phosphorylation and isomerization of the molecule produce isopentenyl pyrophosphate (IPP) and dimethylallyl pyrophosphate (DMAPP). Further condensation of two IPPs to DMAPP generates FPP. FPP is then used for cholesterol synthesis and also acts as a cytosolic covalent messenger for protein prenylation (farnesylation) via the action of farnesyl transferase enzymes. Different from this enzymatic farnesylation mechanism, TRPV3 is likely activated by external FPP without help from cytosolic enzymes [161].

Nonpolar isoprene repeats and polar phosphate moieties are both structurally required for TRPV3 activation, reminiscent of the amine conjugate cases for TRPV1 [161]. FPP was useful to determine TRPV3’s nociceptive role. TRPV3 is expressed primarily in the epidermis rather than in C-fibers, and a relatively mild heat threshold has often cast doubt on its role for pain. *In vivo* FPP treatment assays have clarified the contribution of TRPV3 to inflammatory thermal pain [161]. This FPP-evoked nociception is readily mitigated by TRPV3 knockdown. However, the amount of FPP synthesized in tissues neighboring TRPV3-(+)-epidermal keratinocytes or nerve fibers under a chronic pain state still needs to be explored. Nitrogen-containing bisphosphonates, which are prescribed for osteoporosis, directly inhibit FPP synthase enzyme, resulting in reduced FPP production [162]. These congeners are effective in certain types of neuropathic pain and bone cancer pain [163,164]. Thus, it would be interesting to determine whether a diminished FPP-TRPV3 interaction constitutes an analgesic mechanism. In addition, it would be hypothesized that a deviated mevalonate metabolism affects skin or mental health since TRPV3 is shown to play a role in epidermal pathophysiology and emotional regulation [165,166,167,168].

DMAPP, the precursor of FPP, activates and sensitizes TRPV4 [169]. DMAPP is an activator that is 10 times weaker than EETs. DMAPP is known as a non-peptide phosphoantigen that T cell receptors of γ9δ2-bearing T lymphocytes recognize [170,171]. Since local DMAPP injections in mice lead to nocifensive behavior that is too rapid to be explained by T cell infiltration, peripheral TRPV4 may directly mediates this effect [169]. As mentioned above, mechanical phenotypes are more readily detected compared to thermal phenotypes *in vivo* when TRPV4 is manipulated. Consistently, DMAPP injections only sensitized mechanical but not noxious heat-induced behaviors. Like FPP, however, the level of endogenous DMAPP produced near the TRPV4-expressing nerve termini in chronic pain states remains elusive. Micromolar levels of DMAPP, which are sufficient to open TRPV4, have been predicted to exist in plants and humans [172,173,174]. Leakage of intracellular contents as a result of physical tissue damage might be a plausible situation for elevated DMAPP levels and perhaps FPP as well.

Retinoids contain isoprene repeats, but do not share a synthetic metabolism with the cholesterol precursors described above in the body. Instead, retinoids are generated from carotenoid breakdown and further alcohol and aldehyde dehydrogenation. Members of the retinoid family have been shown to be important in properly maintaining rhodopsin structure, neuronal outgrowth and differentiation, and restoring neuronal function after injury. Among endogenous retinoids, all-*trans*-retinoic acid and 9-*cis*-retinoic acid selectively activate TRPV1 [175]. These substances appear to bind to the capsaicin binding site, which is not consistent with the binding site prediction for FPP. Likewise, their ring combined with a carbon chain reminds of the capsaicin chemical structure. It is tempting to speculate that an aberrant retinoid metabolism may alter TRPV1-mediated nociception, as many neuronal disorders associated with its metabolism have already been reported, although nuclear receptors are likely responsible for the previous cases [176].

#### 3.2.5. Covalent Ligands on TRPA1

Throughout this review, the binding mode of lipids seems to be a non-covalent manner as known from typical second messenger interactions and drug-ligand interactions. TRPA1 offers a unique exception: TRPA1. Although the TRPA1 ligand hepoxilin was briefly introduced above as a non-covalent activator, TRPA1 is likely entrusted with detection of reactive substances via its covalent binding potential [177,178]. Curiously, the critical covalent binding site of TRPA1 is located in the cytosol (*N*-terminal nucleophilic cysteine, lysine or histidine residues) despite the approach of numerous ligands from outside the cell. Space constraints within the extracellular region may reduce the possibility for contacting these substances. Consistently, covalent TRPA1 ligands have two distinct features: the sufficient lipophilicity to permeate the lipid bilayer and access the intracellular nucleophilic amino acids, and electrophilicity to form a covalent conjugation with these residues. Typically, long fatty acyl chains for lipophilicity and at least one highly reactive αβ-unsaturated carbonyl moiety appears to satisfy these two conditions. The αβ-unsaturated carbon of the ligand is nucleophilically attacked by an *N*-terminal cysteine (sulfhydryl group), lysine (ε-amino group), or histidine (imidazolyl group), resulting in covalent bond formation or namely, Michael addition. Well known examples of such lipidergic ligands are the cyclopentenone prostaglandins (PGs): 15-deoxy-Δ12,14-PGJ_2_, and Δ12-PGJ_2_, PGA_1_, PGA_2_, and 8-iso-PGA_2_, [179,180,181,182] (Figure 3).

In fact, covalent interactions between reactive ligands and receptors have been reported previously. With respect to cyclopentenone PGs, they act on nuclear receptors such as peroxisome proliferator-activated receptors (PPARs) or nuclear factor-κB (NF-κB) in a similar covalent manner. Cyclopentane PGs that lack an αβ-unsaturated carbon have no direct effect on TRPA1 or nuclear receptors. Instead, they act on their own receptors such as EP or DP type GPCRs in a typical non-covalent fashion. On the other hand, reactive cyclopentenone PGs do not interact with the GPCRs. It might be possible that these two PG species diverged evolutionarily to regulate different biological processes. All PGs share a common involvement in amplifying inflammatory and nociceptive signals, which is their most important protection mechanism in the body. One difference is their time resolutions. TRPA1 activation by cyclopentenones seems to be an acute alert sign (seconds to minutes), whereas cyclopentanes likely play a relatively long term role (hours to days) for development of inflammation and chronic pain.

Nitrative fatty acids are also covalent TRPA1 activators [183]. Nitric oxide (NO), which is generated during inflammation, participates in diverse inflammatory reactions [184]. NO binds to membrane lipid bilayer components, forming nitrated phospholipids and fatty acids. Nitro-oleic acid was shown to activate TRPA1 via covalent binding of its nitrated carbon with TRPA1 protein. In fact, NO itself also has the potential to directly activate TRPA1 and TRPV1 [185]. Because nitro-oleic acid does not activate TRPV1, but activates TRPA1 more potently than NO donors, NO dissociation is unlikely to mediate the action of nitro-oleic acid [183]. Moreover, an NO scavenger failed to prevent TRPA1 activation. The highest potency (1 µM) among those of endogenous activators ever found for TRPA1 may lead future attention to its physiological importance.

The Michael addition mechanism may also apply to fatty aldehydes containing αβ-unsaturated carbons generated upon oxidative stress: 4-oxononenal (4-ONE), 4-hydroxynonenal (4-HNE), and 4-hydroxyhexenal (4-HHE). These ligands are degraded metabolites of hydroperoxy PUFAs such as HpETEs. While HpETEs are known to be specific to TRPV1, fatty aldehydes activate TRPA1 [179,186,187]. Further, 4-ONE exhibits the highest potency for opening TRPA1 among three fatty aldehydes [179,188], which has been suggested to be because 4-ONE is most electrophilic owing to its two carbonyl moieties [189,190]. During oxidative stress, 4-ONE and 4-HNE are detected at micromolar and millimolar levels at which they open TRPA1, causing pain [191,192,193,194]. Since the fatty aldehydes are more chemically stable compared to HpETEs, they might travel farther from the original oxidative focus, and affect the pain state at different tissue sites [191,195].

EET-TRPA1 interaction in the spinal presynapse was mentioned above. The same study proposed that the covalent binding mechanism explains this interaction, which was elucidated using a TRPA1 mutant lacking critical cysteine/lysine residues [157]. Indeed, cysteine interaction with lipid epoxides has been previously demonstrated. For example, opening of the epoxide ring of LTA4 via the reaction between cysteine residue of glutathione transferase and the epoxide carbon of the lipid is the critical step for its enzymatic conversion to LTC4 [196]. Accordingly, it also seems to be required to revisit the TRPV4 interaction mechanism with EETs.

TRPA1 specificity of the covalent lipids remains a matter of some debate. The De Groat group showed that nitrooleic acid can also activate TRPV1 and even other unknown ion channels [197,198]. 4-ONE has also been shown to activate TRPV1 at high concentrations. These blurred results could be due to decreased selectivity, which is an innate feature of Michael addition. As mentioned above, nucleophilic amino acids are cysteine, histidine and lysine. Those are not specific components of TRPA1 but are present within the sequences of all ion channel proteins, including TRPV1. Thus, it is possible that Michael addition occurs not only in critical *N*-terminal residues, but also in many other regions. However, the covalent reactions of those critical target sequences may have a stronger conversion potential turning their structural changes into an allosteric pore opening. Different reactivities of multiple target amino acids may enable concerted and graded outcomes of changes in the open probability, as seen in typical dose-response curves. The TRPA1 protein is likely the most sensitive TRP for this chemo-electrical conversion, while TRPV1 protein could have a low efficiency.

### 3.3. Membrane Incorporation Hypothesis?

When exposed to a lipophilic molecule, the lipid bilayer of the cellular membrane can experience changes in its fluidity or thickness, possibly affecting ion channel structure and gating [99,199]. Mechanosensitive channels appear to be more vulnerable to these changes, and have been shown to be activated or sensitized by application of PUFAs [200,201,202,203,204]. Decreased membrane stiffness and a thinner bilayer structure upon PUFA incorporation have been proposed as the mechanism responsible for conferring the ready opening of these channels. The cholesterol-rich bilayer architecture in a specialized region of the plasma membrane may also affect the membrane properties, which has been reported to alter temperature thresholds of TRPM8 and TRPV1 openings [205,206,207]. Different from this view, Picazo-Juárez *et al.* once suggested a specific cholesterol-binding site in the transmembrane (TM) 5 region, a little behind that for capsaicin [208].

Using membrane cupformer and crenator assays, Vanden Abeele *et al.* suggested that lyso-phospholipids (LPLs) may locally alter the bilayer micro-curvature, thereby positively contributing to TRPM8 opening [209]. However, further studies are needed to conclude the activation mechanism since TRPM8 is recalcitrant to a mechanical stretching. Other possibilities also remain to be tested including direct GPCR activation by LPL, which was once proposed for TRPV2 [210]. LPLs refer to structurally similar lipids but have a variety of roles in apoptosis, immune regulation, atherosclerosis, and other processes. With respect to these biological roles, members of lysophosphatidic acid (LPA)-activated receptor family are well defined but sugar or amine-conjugate forms, including lysophosphatidyl choline (LPC), lysophosphatidic inositol (LPI) and lysophosphatidyl serine (LPS), are still undergoing deorphanization and appear to be heterogeneous (toll-like receptors, GRCR, and ion channels and so on). Interestingly, all three of these substances activate TRPM8 [209,211,212]. These LPLs also heighten TRPM8 cold sensitivity.

Ca^2+^-independent PLA2 (iPLA2) is known to constitutively produce LPLs. Accordingly, LPLs appeared to be fascinating but unlikely molecules in terms of the on-demand release that is required of intercellular transmitters or a second messengers. Later, however, the stimulant-dependent *de novo* generation of LPL by cytosolic cPLA2 was demonstrated and was also found to be coupled with TRPM8 activation [213]. Nonetheless, it is still disputed whether membrane component theory can commonly explain lipid-TRP interactions as a specific binding mechanism is being strongly suggested for other TRP cases as described below. The fact that a limited number of lipids act on sensory TRP channels and that the outcomes are varied such as activation, sensitization, and inhibition also possibly exclude the membrane integrity mechanism and very probably a nonspecific detergent effect via bilayer partitioning.

Core properties extracted from their chemical structure of lipidergic ligands might help to demonstrate how lipids and TRPs specifically interact. Although a thorough exploration of the basis of how long carbon chains are optimal for TRP binding and gating, recent studies have suggested that aliphatic chain length may determine TRP activation potency. Longer chains appear to elicit bigger responses from TRPA1 [214,215]. Similar features were once reported in a study using a TRPM8-activating lipid LPC (16:0) and an analogue with a different length (6:0) [211]. For TRPV3, fatty acids with a length of 22 or 18 carbons showed lower efficacy than those with 20, indicative of an optimal range [137]. Also, a larger number of unsaturated bonds tend to derive a greater response [87,137]. Probably the location of double bond is also important as numerous TRP ligands were identified among n-3 and n-6 PUFAs. This double bond location theory is also true for *trans*-fats since elaidic acid and linoelaidic acid have been shown to activate *Drosophila* TRPL and to sensitize TRPV3 [85,137]. Again, *trans*-isomers are well known not to increase bilayer fluidity, and TRP modulation by lipid may not be mediated by membrane incorporation. It remains to be seen how 3-dimesional twists caused by polyunsaturation contribute to the formation of an optimal shape that fits the putative binding pocket.

Amphiphilic structures comprising a relatively polar moiety and an aliphatic chain are frequently found in lipidergic TRP modulators such as fatty acid amine conjugates, phosphoryl isoprenoids and even among exogenous plant substances such as the canonical TRPV1 activator capsaicin. In addition, in an energy-minimized 3-dimensional view, relatively polar region composed of hydroperoxy residues in HpETE species was found to contribute to forming capsaicin-like structure [87]. Again, this amphipathic property is reminiscent of the cellular membrane bilayer. Ruling out a nonspecific detergent effect, the lipid binding pocket might be located near the TM domains, and a specific bilayer interface might constantly contact this region at rest. This resting interaction could be intervened by lipidergic ligands having an optimized size and shape, which may lead to allosteric changes in the channel pore structure. This appears to be true at least for TRPV1. Very recently, the structure of TRPV1 protein is defined using electron cryo-microscopy [216,217]. Capsaicin binds to a region above E570 in the S4–S5 linker (TM4–TM5 linker) and L669 in TM6. In voltage-gated channels, the S4–S5 linker is generally important in converting S4 voltage sensor movement upon depolarization into pore gating. Thus, in the case of TRPV1, a similar allosteric mechanism mediated via its S4–S5 linker likely works between ligand binding and TRPV1 opening. Surprisingly, this binding pocket is occupied by an unidentified lipophilic molecule in a resting state [216], indicating that TRPV1 ligands probably replace an endogenous pre-occupying inert lipid (Figure 1). It remains to be seen whether this mechanism is conserved in other TRP-lipid interactions.

### 3.4. Silencing Ligands or Negative Modifications

Intracellular signal transductions initiated by plasma membrane receptor-ligand interactions frequently consist of amplification of signals via boosting downstream enzyme cascades or ion channel activities. Sometimes, inhibitory modulations on enzymes or ion channels also occur such as Gi-coupled reduction in cAMP formation. Likewise, and even more notably, when we see the top upstream, the receptor-ligand interaction, it appears to be based on agonism, while competitive antagonism or inverse agonism appears to be relatively rare, and may instead be an artificial or strategic phenomenon to manipulate pathologic conditions [218]. In nature, inhibitory modulations seem to be accomplished primarily by control of agonist synthesis/secretion/reuptake or by tuning receptor sensitivity and numbers. Nonetheless, an increasing number of studies have demonstrated ligand-receptor antagonism, particularly in ion channel fields. Indeed, K^+^ channel studies opened the field by presenting lipidergic endogenous inhibitors [219,220,221].

In the sensory TRP channel field, adenosine was the first reported endogenous TRPV1 inhibitor [222]. For endogenous lipidergic substance action on the sensory TRPs, anandamide and NADA, which are TRPV1 activators, were first found to inhibit TRPM8 [223]. Interestingly, TRPV1, which is heat sensor, and TRPM8, which is a cold sensor, exhibit opposite features in some regards. Phosphorylation by protein kinase A results in sensitization of TRPV1, but inhibition of TRPM8 [105,223]. PIP2 binding disturbs TRPV1 activation but activates TRPM8 (see above). Thus, it is tempting to explore whether opposite and parallel axes for those two TRPs are well conserved in other unknown modulatory mechanisms.

Lipids generated from monocytes/macrophages, injured tissue and the vascular endothelium comprise the major regulators dominating the progression of acute inflammation. Lipidergic regulators can be subcategorized into two classes according to the inflammatory phases, namely, pro-inflammatory and resolving classes [224,225]. n-6 Derivatives including PGs and leukotrienes are primarily responsible for developing inflammation (pro-inflammatory). n-3 Derivatives like resolvins or maresin act as resolvents. While production of both pro-inflammatories and resolvents is initiated as soon as injured or inflamed, the time for the peak tissue levels of resolving lipids is hours or days behind that of pro-inflammatory lipids, which determines the duration of the inflammatory state (typically one to several days). Thus, this indicates that the progress and severity of inflammation likely depends on the balance between pro-inflammatory and pro-resolving mechanisms. When the resolution is unsuccessful, chronic inflammation occurs. Interestingly, there are similarities in the role of lipids in inflammation and pain. n-6-Derived pro-inflammatory lipids such as PGs and HpETEs activate sensory TRPs, thereby eliciting pain sensation. In contrast, n-3 lipid mediators prevent or relieve pain: part of this mechanism is antagonism by resolvins and neuroprotectins of sensory TRP channels.

**Figure 4:**
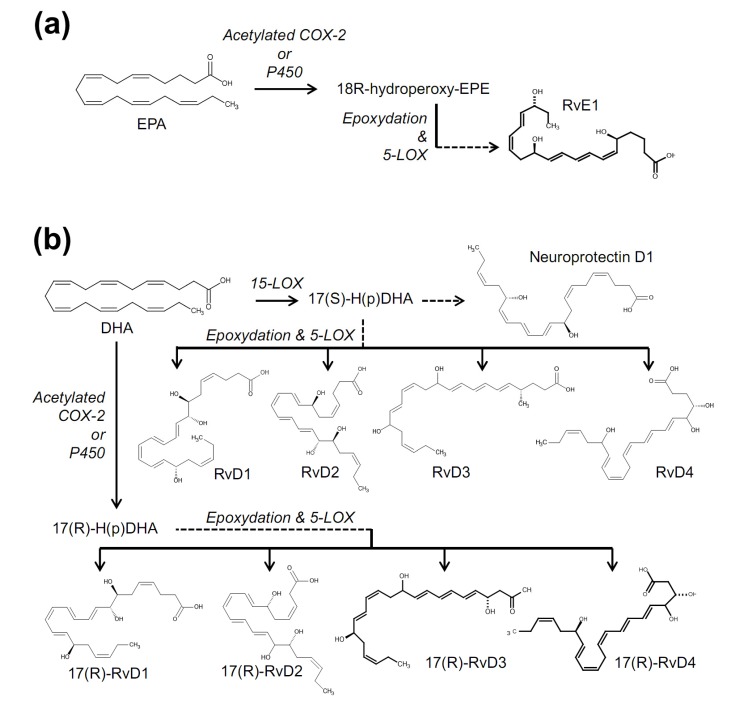
Biosynthetic pathways for resolvin E1 (RvE1) (**a**); and resolvin Ds (RvDs) (**b**).

LOXs of monocyte/macrophage and neutrophil metabolize n-3 fatty acids (docosahexaenoic acid {DHA} and eicosapentaenoic acid {EPA}) into resovin D and E species (Figure 4). D and E represent their source precursors, DHA and EPA respectively. Among the approximately 30 subtypes of resolvin molecules known, six lipids have been tested in terms of sensory TRP-mediated actions [226]. 17(*R*)-resolvin D1 is a specific inhibitor for TRPV3 [227], thereby attenuating TRPV3-mediated pain phenotypes. In this way, similar to the case of FPP, this substance was useful to show that TRPV3 mediates a type of heat pain modality. 17(*S*)-resolvin D1, an enantiomer of the 17(*R*) form, exhibits broader inhibitory actions on TRPA1, TRPV3 and TRPV4 at nanomolar and micromolar levels [228]. Low specificity and high potency seems to confer a good analgesic efficacy against in a multitude of animal pain models *in vivo*, even when administered locally. In addition, resolvin D2 inhibits TRPV1 and TRPA1. Resolvin E1, neuroprotectin D1 and maresin 1 (macrophage mediator in resolving inflammation 1; a metabolite of DHA) inhibit TRPV1 [229,230,231,232]. Resolvins are known to activate their primary metabotropic receptors such as chemR23 and GPR32 when they act on immune cells [224]. C-fibers has been shown to express these GPCRs and inhibition of TRPs may result from G protein mediated indirect signal transductions although D1 enantiomers described above are unlikely to utilize these pathway based on the G protein inhibitor assays.

The strong potencies of these resolving lipids at nanomolar levels that were commonly observed in TRP studies appear to make their future utility promising in the pain management field. In the inflammation field, these substances are already shown to strongly resolve varied types of inflammatory diseases without adverse effects in animal models. Not only administration of resolvents or their synthetic analogues, but also promoting their production in tissue by manipulating the function of their metabolic enzymes could be attractive therapeutic strategies [225,226].

By and large, sensory TRPs seem to discern n-6 and n-3 unsaturated fatty acids as their activators and inhibitors, respectively. An exception is TRPM8: it is inhibited by either type of the PUFAs [211]. Both TRPM8 activation in response to cold temperatures and menthol are attenuated in the presence of EPA, DHA, or arachidonic acid. On the other hand, arachidonic acid activates TRPM2, the closest paralog of TRPM8 [233]. It will be interesting to know which of the unconserved amino acids determine this differential gating.

Micromolar IPP inhibits TRPA1 and TRPV3 and suppresses pain modalities mediated via these two TRP channels [234]. Its pyrophosphate moiety is important for both inhibitory properties and TRP selectivity. As mentioned, IPP is an intermediate molecule in the HMG-CoA reductase pathway. FPP has been shown to act on the external surface of TRPV3, and IPP blocks this effect in a reversible manner. Therefore, IPP and FPP might share binding sites somewhere in the extracellular region of TRPV3. IPP levels in our body are not well-known. IPP level might be considered to be around 1–3 fold higher than the level of its metabolite FPP, which was be detected at nanomolar ranges. Thus, IPP is presumably present in the range of tens of nanomoles [172]. It might be interesting to ask whether the level is regulated up to levels needed for controlling TRPs, in certain disease conditions. It might also be interesting to determine whether pain states can be improved by controlling IPP production or its metabolizing enzymes. Cells accumulate IPP in the cytosol when treated with bisphosphonates [235]. Thus, it might be worth revisiting the analgesic mechanism of bisphosphonates regarding the IPP-TRP information.

Although IPP has a relatively simple chemical structure, it has been studied as an exogenous antigen in the immunology field, like DMAPP. The human T cell receptor of γ2δ2 T cells detects exogenous IPP [170], which may initiate T cell activation and subsequent immunologic cascades. It remains unclear whether this immune mechanism is independent of the sensory TRP detection of IPP or whether those mutually interact and are co-evolved as a defensive mechanism.

## 4. Conclusions and Perspectives

As soon as sensory TRP channels were discovered in the late 1990s, their interactions with lipids began to be noticed. Since then, sensory TRP research has generated a series of interesting hypotheses. In turn, in the process of examinations of those hypotheses, TRP-mediated pain mechanisms are being elucidated. This review provided an overview of the roles for individual sensory TRP channels in nociception and summarized the endogenous lipids that act on TRPs mostly in the context of pain modulation (Table 1). Recent findings of the molecular mechanisms of their interaction were also introduced. Based on the knowledge, we need to proceed to answer a large number of unsolved problems.

**Table 1:** The list of endogenous lipids that regulate sensory TRP channel activities introduced in this review and their TRP channel targets.

Sensory TRP channels	Endogenous lipidergic activators	Endogenous lipidergic inhibitors
TRPA1	Arachidonic acid, DAG [64], PIP2 [120,121] Cyclopentenone prostaglandin (PGs) [179,180,181,182] Nitro-oleic acid [185] 4-ONE, 4-HNE, 4-HHE [179,186,187]	17(S)-resolvin D1 [228] Resolvin D2 [231]
TRPV1	12(S)-HpETE, 15(S)-HpETE [87,103] oxoODEs, HODEs [111] DAG [112,113] NADA, OLDA, OEA, retinoic acid [86,126,128,129,130]	Resolvin D2 [231] Resolvin E1, neuroprotectin D1, maresin 1 [229,230,231,232]
TRPV3	arachidonic acid [137] FPP [161]	17(R)-resolvin D1 [227] 17(S)-resolvin D1 [228]
TRPV4	EETs [138,151] DMAPP [169]	17(S)-resolvin D1 [228]
TRPM8	PIP2 [108] LPC, LPI, LPS [209,211,212]	EPA, DHA, arachidonic acid [233]

Because of their diffusible nature and binding mode that is not yet completely understood, it is still less clear whether individual lipids conform with the classical neurotransmitter binding, second messenger modulation, or membrane bilayer incorporation concepts, although those are roughly categorized here. This has not been an issue when metabotropic prostaglandin receptors, LPA receptors, or cannabinoid receptors were found. Their lipids bind to an external region of the receptors, which initiates internal enzymatic signaling cascades. Indeed, the heterogeneity of interaction modes predicted for TRP channels thus far require a more concerted effort to accumulate information about the molecular properties of each lipids and TRP proteins. Accompanying structural information will also narrow down leads for analgesics aimed at inhibiting TRPs.

Vesicular transport of TRP-modulating lipids is considered improbable. It is unknown how rapidly lipids with a polar residue permeate the plasma membrane of excretory and receiving cells. The production processes of some lipids also remain elusive. For example, the activity of PLA2 and PLC are coupled to activation of membrane metabotropic receptors. However, it is unclear whether the downstream enzyme LOX is activated, overexpressed or translocates to the membrane upon relevant stimuli, or if it is otherwise tonically active. Attention to the source substrates (DHA, EPA, *etc.*) for these enzymes is increasing in the nutritional and cardiovascular fields. However, it remains blurred how much the levels of these substrates fluctuate inside our bodies. Nutritional supply may be a critical factor controlling substrate levels, and may affect the pain or inflammatory states [236,237].

In conclusion, sensory TRP channels are crucial molecular components for peripheral pain mediation. Research on TRP modulation by lipidergic endogenous substances has greatly extended our knowledge of the initiation and exacerbation of pain. Future exploration of the lipid-TRP interaction will contribute to a better understanding of the molecular mechanisms of pain and may also help in devising pharmacological strategies for manipulating pain.
